# Taste recognition through tarsal gustatory sensilla potentially important for host selection in leaf beetles (Coleoptera: Chrysomelidae)

**DOI:** 10.1038/s41598-020-61935-x

**Published:** 2020-03-18

**Authors:** Shun Yosano, Yasuhiko Kutsuwada, Minami Akatsu, Shuhei Masuta, Rei Kakazu, Naoshi Masuoka, Kazuhiro Matsuda, Masatoshi Hori

**Affiliations:** Graduate School of Agricultural Science, Tohoku University, Sendai, Miyagi 980-8572 Japan

**Keywords:** Chemical ecology, Entomology

## Abstract

It is well known that Diptera and Lepidoptera can recognize tastes through their legs, which allows them to select suitable hosts. In Coleoptera, the largest insect order, however, the role of the legs in taste recognition to aid in host selection is unclear. In the present study, we investigated taste recognition through the legs of Chrysomelidae, Coleoptera. Through morphological observations, we found that all subfamilies of Chrysomelidae exhibit gustatory sensilla in the distal leg segment, i.e., the tarsus. In contrast, we did not find evidence of these sensilla in the species that we examined from four families of Coleoptera. We confirmed that different tastes, i.e., sweet, bitter, and leaf surface wax, were received through the tarsal sensilla of Chrysomelidae by recording the electrophysiological responses of the sensilla. Further, we found that *Galerucella grisescens* (Chrysomelidae) can respond to different tastes used in the electrophysiological tests using only their tarsi, whereas *Henosepilachna vigintioctomaculata* (Coccinellidae), lacking tarsal gustatory sensilla, did not exhibit similar responses. Our results suggest that although tarsal taste recognition is not common throughout Coleopteran species, it may be a common feature in Chrysomelidae, and tarsal gustation may play an important role in host selection in this family.

## Introduction

Gustatory organs can be found in different body parts of an insect. In addition to their mouthparts, insects are able to recognize taste with their antennae, legs, ovipositor, and/or wings^1–5^. Taste recognition through gustatory sensilla on the tarsus (distal leg segment), is well studied, especially in Diptera and Lepidoptera. In these orders, it has been revealed that several tastes including sweet, bitter, and sour are recognized through their tarsi^5–9^. The tarsi are the first body part to come into contact with the surface of a plant as an insect lands; therefore, tarsal gustation is important for insect host selection. Butterflies drum on the surface of leaves after landing on them to evaluate their suitability as an oviposition site^10,11^. Diptera recognize phagostimulants, such as sucrose, with their tarsi^12,13^. In addition, contact sex pheromones are detected through the tarsi^14–16^; thus, tarsal gustation is important for mating. Also, tarsal gustation is reported in other orders; for example, honeybees (*Apis mellifera*, order Hymenoptera) show an appetitive response to sucrose tasted through their tarsi^17,18^. In addition to morphological, behavioural, and electrophysiological studies, the expression of gustatory receptors involved in tarsal gustation is well studied in Diptera and Lepidoptera^19–21^.

Tarsal taste recognition in Coleoptera (beetles), however, remains poorly understood, even though it is the largest order of insects. The presence of tarsal gustatory sensilla is reported in a limited number of coleopteran species, such as *Callosobruchus maculatus* and *C. subinnotatus* (Coleoptera: Chrysomelidae)^22^. Thus, it is still unclear whether tarsal gustatory sensilla are common in Coleoptera. In some species, such as *Tribolium castaneum* (Coleoptera: Tenebrionidae), expression of tarsal gustatory receptor proteins has been demonstrated; however, the role of these receptors in taste recognition is not fully understood^23^. Similarly, although it has been demonstrated, using electrophysiological methods, that the tarsal gustatory sensilla observed in *Chrysolina brunsvicensis* (Coleoptera: Chrysomelidae) receives hypericin, a compound specific to its host plant^24^, the association between tarsal taste reception and behaviour is still unknown. The role that tarsal taste recognition plays in coleopteran host selection is, therefore, also poorly understood.

In this study, we focused on tarsal taste recognition in Chrysomelidae because this family includes many important agricultural pests. To clarify the presence and the role of tarsal taste recognition in Chrysomelidae, we investigated the role of tarsi as gustatory organs through morphological, electrophysiological, and behavioural experiments. In these experiments, we used the strawberry leaf beetle, *Galerucella grisescens*, as a representative species of Chrysomelidae because this beetle can be easily reared, and chemical substances involved in the feeding and host orientation of this beetle have previously been identified^25–28^. We demonstrate that tarsal gustatory sensilla are common in Chrysomelidae and that this trait may be important for host selection in Chrysomelidae.

## Results

### Tarsal gustatory sensilla in Chrysomelidae

**Figure 1:**
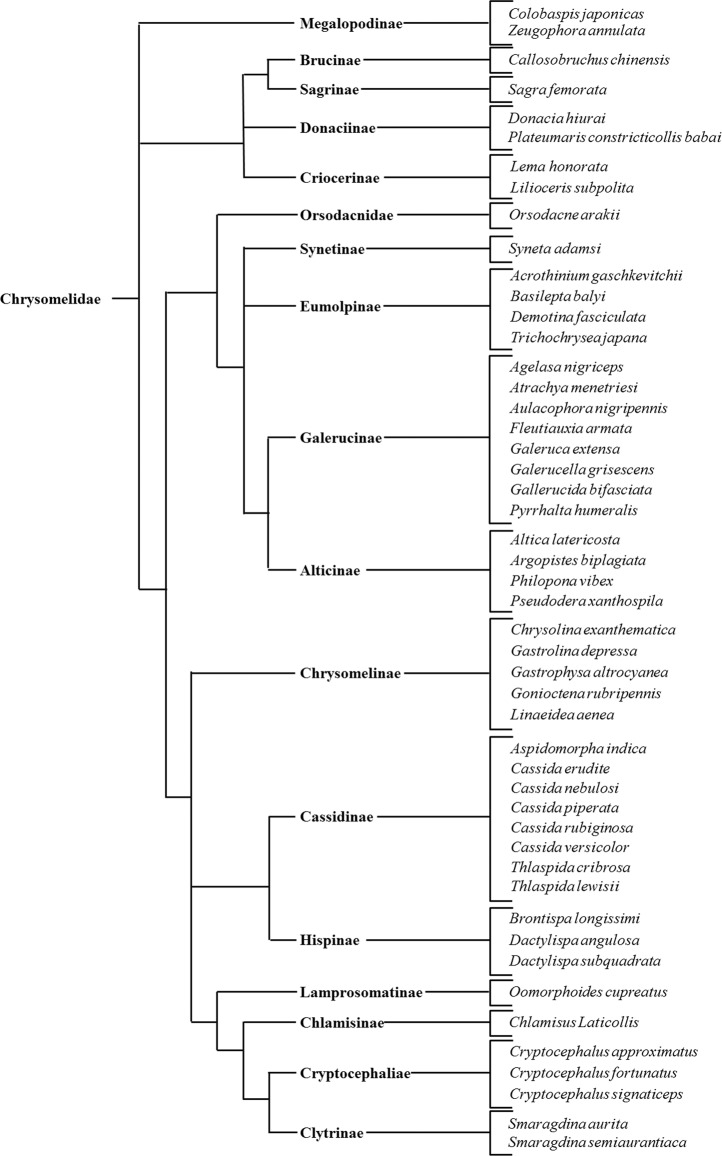
Presence of tarsal gustatory sensilla in Chrysomelidae. All species observed have tarsal gustatory sensilla.

**Figure 2:**
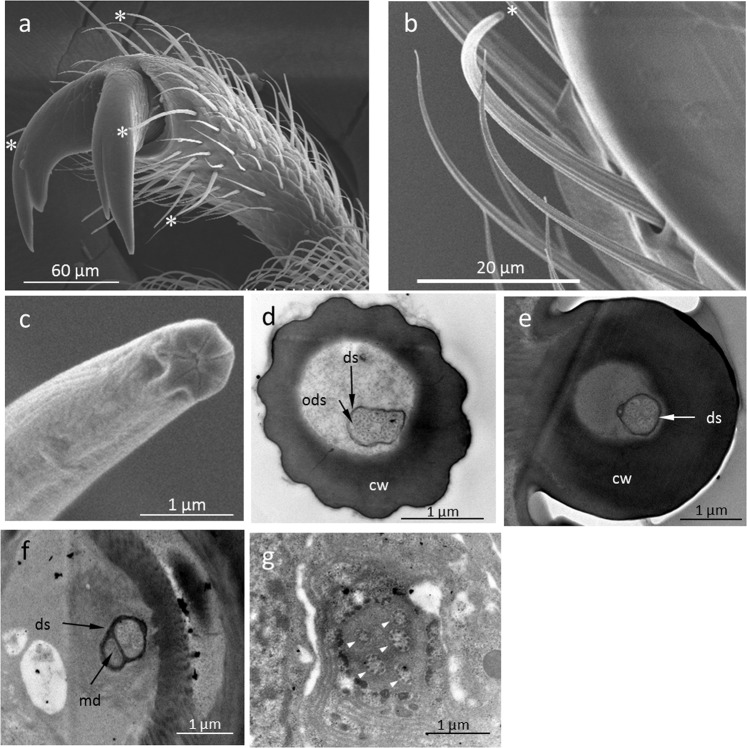
Morphology of a tarsal gustatory sensillum of *Galerucella grisescens*. (**a–c**) Scanning electron micrographs of *G. grisescens* tarsal gustatory sensilla. (**a**) Fifth tarsomere. *Sensillum chaeticum. (**b**) Tarsal sensilla near the claw. *Sensillum chaeticum. (**c**) Tip of the tarsal sensillum chaeticum showing a sensillum pore surrounded by several ridges. (**d–g**) Transmission electron micrographs of *G. grisescens* tarsal gustatory sensilla. (**d**) Cross section of a tarsal sensillum chaeticum at the peripheral level. Four dendrites are enveloped by a dendritic sheath. ods: outer dendritic segment. ds: dendritic sheath. cw: cuticular wall. (**e**) Cross section of the same sensillum at the base level. (**f**) Cross section taken under the socket of the same sensillum. In addition to the four taste dendrites, a mechanosensory dendrite (md) is shown. (**g**) Cross section of the same sensillum at ciliary level. Five sensory neurons were observed (arrowheads).

Insect gustatory sensilla are uniporous bristles with the pore located at the tip^29^ and are identifiable using electron microscopy. To clarify whether tarsal gustatory sensilla are common in Chrysomelidae, we examined the tarsi of 49 Chrysomelidae species (Table S1) under a scanning electron microscope (SEM). We found uniporous tarsal sensilla in all subfamilies of Chrysomelidae (Figs. 1, S1,S2). For example, the tarsal gustatory sensilla of *G. grisescens* have a pore surrounded by several ridges on the tip and those of *Cassida nebulosa* have no ridges on the tip. (Figs. 2a–c, S1). Insect gustatory sensilla contain several dendrites extending from gustatory neurons^30,31^. To understand the internal morphology of uniporous sensilla found on the tarsi of Chrysomelidae, we examined the tarsal gustatory sensilla of *G. grisescens* under a transmission electron microscope (TEM). We observed that the tarsal gustatory sensilla of *G. grisescens* are innervated by four dendrites, which are enveloped by a dendritic sheath (Fig. 2d). These dendrites are considered to be taste neurons as they extend from the ciliary level to the tip of a sensillum (Fig. 2d–g). Also, we found another dendrite extending to the sensillum base, suggesting that these tarsal gustatory sensilla contain a mechanosensory neuron in addition to the gustatory neurons (Fig. 2f,g). A similar characteristic was observed in the internal morphology of uniporous sensilla of *C. nebulosa* (Fig. S5). From these TEM observations, it was revealed that tarsal uniporous sensilla of Chrysomelidae are gustatory sensilla.

**Figure 3:**
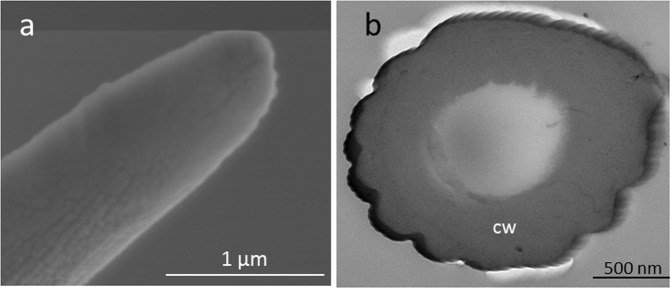
Morphology of a tarsal sensillum of *Henosepilachna vigintioctomaculata*. (**a**) Scanning electron micrograph of a *H. vigintioctomaculata* tarsal sensillum. Sensillum pore was not observed on its tip. (**b**) Transmission electron micrograph of a *H. vigintioctomaculata* tarsal sensillum. No dendrite was observed in the sensillum lumen. cw: cuticular wall.

We found that tarsal gustatory sensilla are common in Chrysomelidae, but it is not clear whether tarsal gustatory sensilla are common in other families of Coleoptera. To investigate this, we examined the tarsi of some coleopteran species from other families, namely, *Allomyrina dichotoma* (Coleoptera: Scarabaeidae), *Tenomerga mucida* (Coleoptera: Cupedidae), *Ancylopus pictus* (Coleoptera: Endomychidae), and *Henosepilachna vigintioctomaculata* (Coleoptera: Coccinellidae) using SEM. We found no tarsal gustatory sensilla on the tarsi of any of these species (Fig. 3a). Additionally, we examined the internal morphology of the tarsi of *H. vigintioctomaculata*, which is phytophagous like Chrysomelidae. We found no dendrites inside the tarsal sensilla that were present in *H. vigintioctomaculata* under the TEM (Fig. 3b). Thus, we found no evidence for tarsal gustatory sensilla in these four families of Coleoptera, based on the species we examined. This may suggest that, although tarsal gustatory sensilla are common in Chrysomelidae, they are not common throughout Coleoptera.

### Taste reception in the tarsal gustatory sensilla of Chrysomelidae

It has been shown in previous studies that an insect gustatory sensillum can receive several tastes, such as salty, sweet, and bitter^32^. To confirm the taste reception by tarsal gustatory sensilla in Chrysomelidae, we investigated the neural response of tarsal gustatory senisilla of *G. grisescens*. We used a tip-recording method^33–35^ to record the electrophysiological response of the tarsal gustatory sensilla to a range of substances with different tastes. We used KCl and NaCl to investigate the response of tarsal gustatory sensillum to electrolytes. Sucrose is a common phagostimulant for insects including phytophagous species, such as those in Chrysomelidae. Bitter substances detected by gustatory sensilla modulate the positive response of insects to tastants^36,37^. We, therefore, investigated the reception of the bitter tasting brucine by tarsal gustatory sensilla. Also, to investigate the response to specific host-plant compounds, we recorded the electrophysiological response of the sensilla to the leaf surface wax of *Rumex obtusifolius*, a host plant of *G. grisescens*, because leaf surface wax is the first substance that an insect comes in contact with when it lands on the plant.

**Figure 4:**
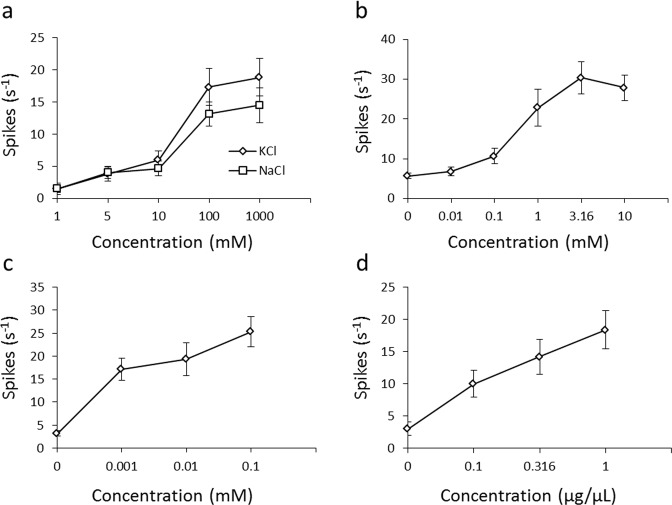
Taste reception by tarsal gustatory sensilla of *Galerucella grisescens*. (**a–d**) Electrophysiological responses of tarsal gustatory sensilla of *G. grisescens*. All recordings were obtained from sensillum C2 using the tip-recording method. Mean spike frequencies (± standard error) generated by applying a solution of each taste substance at each concentration are shown. (**a**) Responses to electrolytes (KCl and NaCl). (**b**) Response to sucrose. (**c**) Response to brucine. (**d**) Response to the leaf surface wax of *Rumex obtusifolius*. Data were obtained from 12 beetles for each tastant.

**Figure 5:**
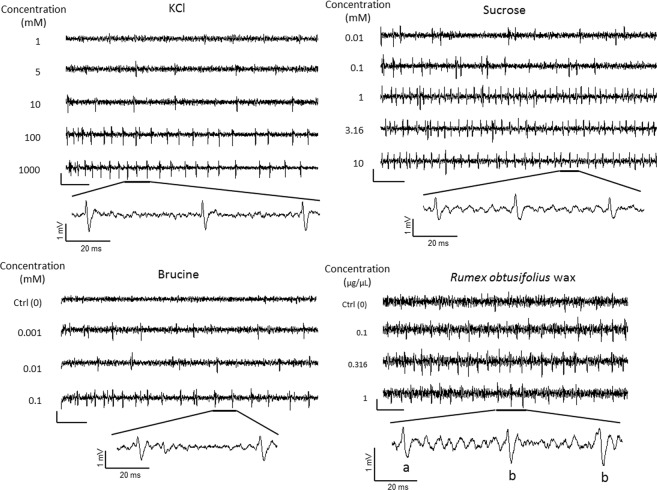
Representative traces of electrophysiological responses obtained from the tarsal gustatory sensilla of *Galerucella grisescens*. Electrophysiological response (waveforms) of the C2 sensillum to the four taste substances (KCl, sucrose, brucine, and leaf surface wax of *Rumex obtusifolius*) obtained using the tip-recording method. KCl, sucrose, and brucine (vertical bar, 1 mV; horizontal bar, 100 ms). Leaf surface wax of *R. obtusifolius* (vertical bar, 400 µV; horizontal bar, 100 ms).

We applied taste substances to three sensillum located on different parts of tarsi (sensilla C1–C3) (Fig. S3). Among three sensilla, only sensillum C2 showed electrophysiological response to all the tastants tested (Fig. 4). KCl and NaCl elicited similar spikes in a dose-dependent manner from the taste neurons of all sensilla (C1–C3) (Figs. 4a, S4a). Sensilla C2 and C3 showed electrophysiological responses to sucrose (Figs. 4b, S4b); however, sensillum C1 showed no response to sucrose. Brucine elicited an electrophysiological response in all the sensilla tested (Figs. 4c, S4c). The leaf surface wax of *R. obtusifolius* elicited an electrophysiological response only in sensillum C2 (Fig. 4d). The different tastants—electrolytes, sucrose, and brucine—clearly elicited different responses from the tarsal sensilla as shown in Fig. 5. The leaf surface wax of *R. obtusifolius* elicited two different spikes [(a) and (b) spikes of *R. obtusifolius* in Fig. 5].

### Tarsal taste recognition in host selection behaviours

In Diptera and Lepidoptera, the presence of tarsal gustatory sensilla is closely related to behavioural responses^8,12^. Therefore, we investigated whether this character had a similar role in Chrysomelidae. To examine the function of tarsal gustatory sensilla, we tested the response of *G. grisescens*, chosen as a model of Chrysomelidae, to sucrose and brucine (20 specimens were used for each test; n = 12). We selected sucrose and brucine to test because these substances elicited electrophysiological responses in the previous experiment. To investigate which gustatory organ is involved in the behavioural response, we compared the responses of beetles in which different gustatory organs (mouthparts, antennae, and/or tarsi) were ablated.

Additionally, we investigated the response of *H. vigintioctomaculata* (10 specimens, n = 12) to sucrose to compare with the tarsal gustatory sensilla in *G. grisescens*. We selected sucrose because it strongly stimulates feeding in this beetle^38,39^.

**Figure 6:**
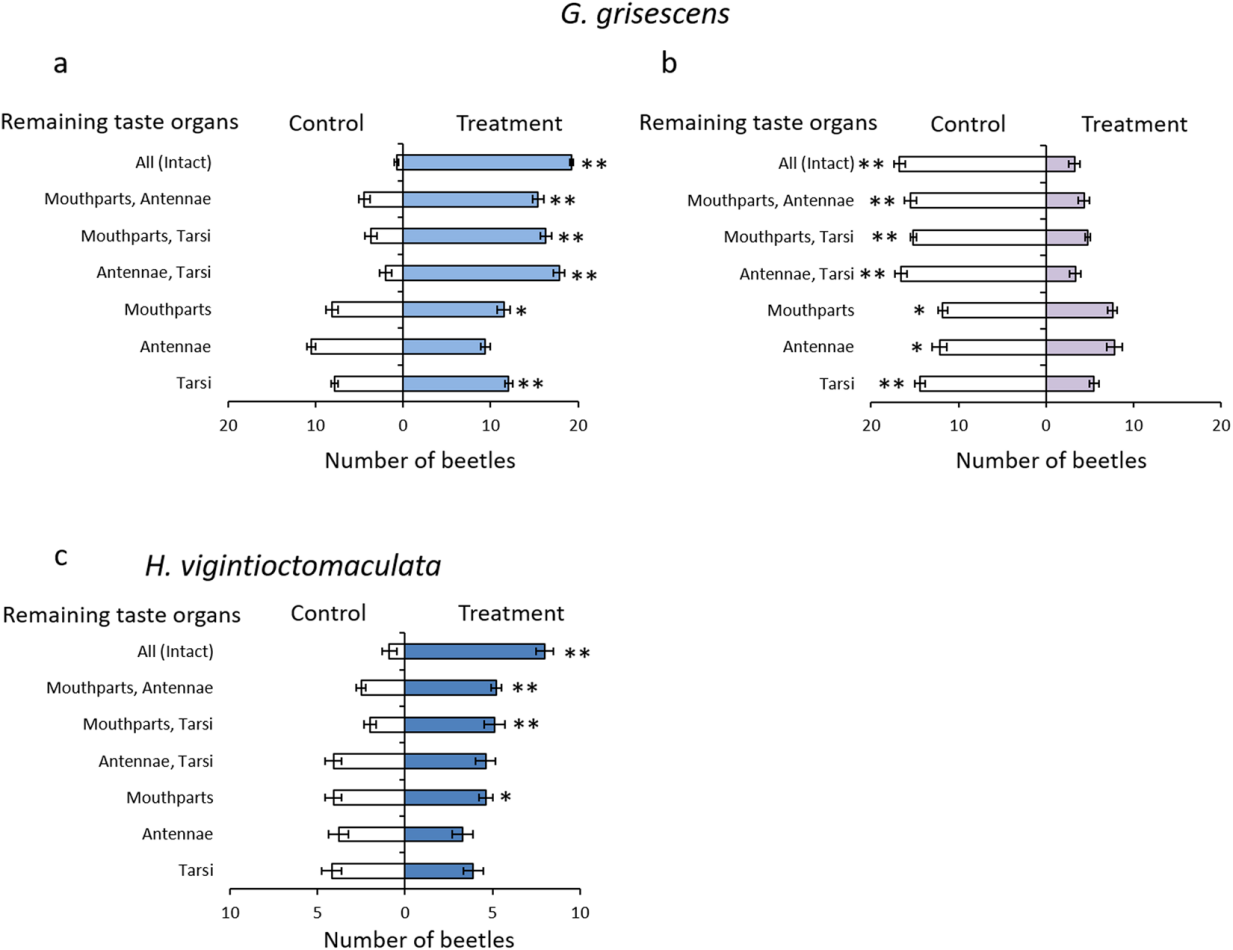
Response of *Galerucella grisescens* to sucrose and brucine and *Henosepilachna vigintioctomaculata* to sucrose. Results of two choice tests for *G. grisescens* and *H. vigintioctomaculata*. (**a–c**) Vertical line shows the gustatory organ that remained after ablation. Horizontal axis shows the mean number of beetles (± SE) that chose the treated or the control half-disk. (**a**) Response of *G. grisescens* to sucrose (20 beetles in each test; n = 12). (**b**) Response of *G. grisescens* to brucine (20 beetles in each test; n = 12). (**c**) Response of *H. vigintioctomaculata* to sucrose (10 beetles in each test; n = 12). A significant difference between the treatment and control is represented by an asterisk (Wilcoxon matched-pairs signed-ranks test: *p < 0.05, **p < 0.01).

Intact (non-ablated) *G. grisescens* showed a positive response to sucrose (Wilcoxon matched-pairs signed-ranks test; *p* < 0.01) (Fig. 6a). *G. grisescens* showed a positive response to sucrose when their mouthparts, antennae, or tarsi were ablated (Wilcoxon matched-pairs signed-ranks test; *p* < 0.01) (Fig. 6a). Also, *G. grisescens* showed a positive response to sucrose tasted using their mouthparts or tarsi only (Wilcoxon matched-pairs signed-ranks test; mouthparts: *p* < 0.05, tarsi: *p* < 0.01) (Fig. 6a). Intact *G. grisescens* showed a negative response to brucine (Wilcoxon matched-pairs signed-ranks test; *p* < 0.01) (Fig. 6b). They showed avoidance after the ablation of mouthparts, antennae, or tarsi (Wilcoxon matched-pairs signed-ranks test; *p* < 0.01) (Fig. 6b). Also, it was found that they could still avoid brucine with only one gustatory system being active (Wilcoxon matched-pairs signed-ranks test; *p* < 0.01 or 0.05) (Fig. 6b). Furthermore, the negative response of beetles with only their tarsi intact was higher than the response of the beetles with only mouthparts or antennae. These results indicate that tarsi are important in recognizing sweet or bitter taste in Chrysomelidae.

In addition, we investigated the behavioural taste response of *H. vigintioctomaculata*, which was found not to possess tarsal gustatory sensilla. Intact *H. vigintioctomaculata* chose sucrose (Wilcoxon matched-pairs signed-ranks test; *p* < 0.01) (Fig. 6c). Their positive response disappeared, however, following ablation of their mouthparts (Fig. 6c). Thus, it was confirmed that *H. vigintioctomaculata* cannot recognize sucrose with their tarsi.

**Figure 7:**
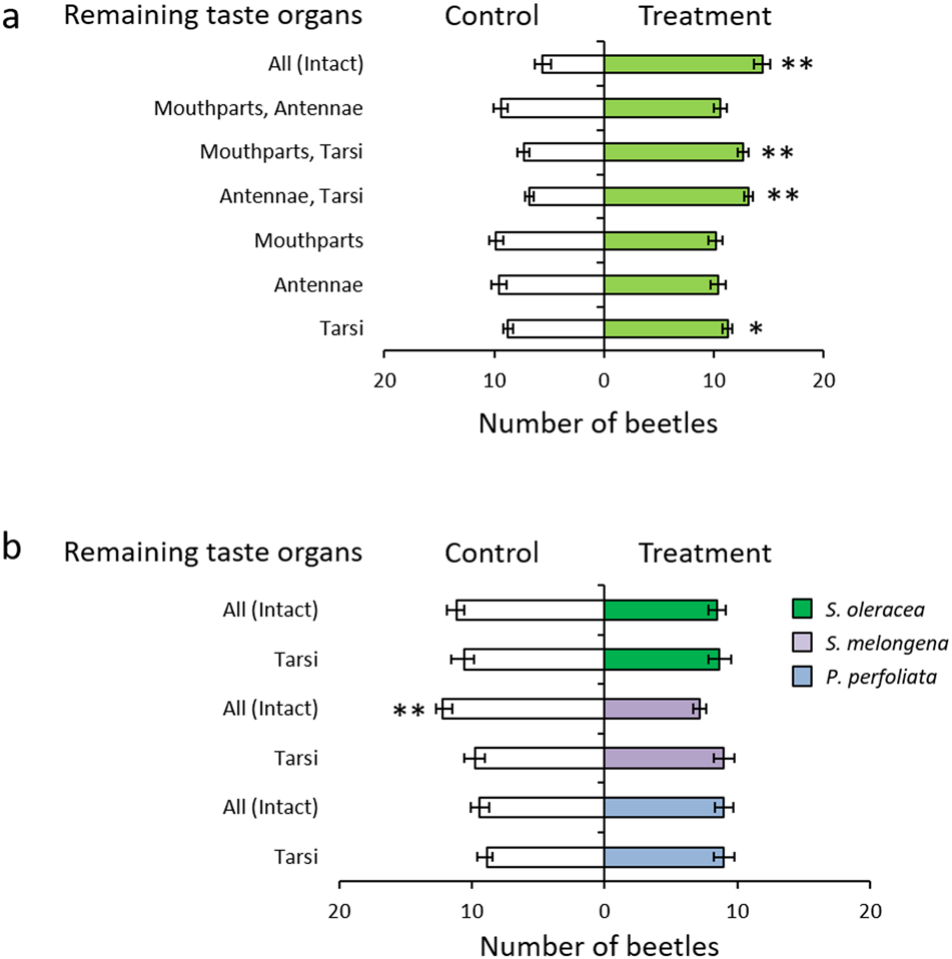
Response of *Galerucella grisescens* to host and nonhost plant leaf surface wax. Results of two choice tests using *G. grisescens*. (**a,b**) Horizontal axis shows the mean number of beetles (± SE) that chose the treated or the control half-disk. Vertical line shows the gustatory organ that remained after ablation. (**a**) Response of *G. grisescens* to leaf surface wax of their host plant, *Rumex obtusifolius* (20 beetles in each test; n = 12). (**b**) Response of *G. grisescens* to leaf surface waxes of nonhost plants (20 beetles in each test; n = 12). A significant difference between the treatment and control is represented by an asterisk (Wilcoxon matched-pairs signed-ranks test: *p < 0.05, **p < 0.01).

The electrophysiological response of insect gustatory organs corresponds to insect behavioural responses to tastants received by these gustatory organs^3,4,40^. Our results are consistent with these studies because *G. grisescens* showed a behavioural response to sucrose and brucine when received through tarsal gustatory sensilla. From these results, it was predicted that *G. grisescens* would also exhibit a behavioural response to the leaf surface wax of *R. obtusifolius*, which also elicited an electrophysiological response when received through the tarsal gustatory sensilla. We tested this by comparing the response of beetles with different gustatory organs ablated to the leaf surface wax of *R. obtusifolius* (20 beetles in each test; n = 12). *G. grisescens* showed a positive response to the leaf surface wax of *R. obtusifolius* when fully intact, and with ablated mouthparts or antennae (Wilcoxon matched-pairs signed-ranks test; *p* < 0.01) (Fig. 7a). They showed a positive response to the leaf surface wax with only their tarsi (Wilcoxon matched-pairs signed-ranks test; *p* < 0.05) (Fig. 7a). Conversely, the beetles did not exhibit a positive response to the leaf surface wax when their tarsi were ablated. The positive behavioural response to the leaf surface wax of *R. obtusifolius* observed in *G. grisescens* with only their tarsi indicates that they can discriminate between host and nonhost plants by tasting leaf surface waxes through their tarsi. We tested this by comparing the responses of beetles to the leaf surface waxes of host and nonhost plants (20 beetles in each test; n = 12) to determine whether they were able to discriminate between them using only their tarsi. We used leaf surface wax of *Solanum melongena*, *Spinacia oleracea*, and *Persicaria perfoliata* as nonhost plants. Unlike the leaf surface wax of *R. obtusifolius*, the leaf surface waxes of *S. oleracea* and *P. perfoliata* did not elicit positive responses in intact *G. grisescens* (Fig. 7b). Also, *G. grisescens* showed no response to these leaf surface waxes with their tarsi only (Fig. 7b). Moreover, intact *G. grisescens* showed a negative response to the leaf surface wax of *S. melongena* (Wilcoxon matched-pairs signed-ranks test; *p* < 0.01) (Fig. 7b), whereas, there was no response in beetles with only their tarsi intact (Wilcoxon matched-pairs signed-ranks test; *p* = 0.6562) (Fig. 7b).

## Discussion

Some insects can taste through specialized tarsal sensilla, but the recognition of taste and associated behavioural responses are poorly understood in Coleoptera. The results from our morphological, electrophysiological, and behavioural experiments demonstrate the role of tarsal taste recognition in the host selection of *G. grisescens* (Chrysomelidae).

We found that members of all subfamilies of Chrysomelidae have tarsal gustatory sensilla. We investigated the internal morphology of tarsal gustatory sensilla using TEM and found that these sensilla, defined as gustatory sensilla by SEM observation, were likely to contain several dendrites regardless of the tip morphology (Figs. 2d, S5). In addition, we found that KCl was perceived by a sensillum that we defined as a gustatory sensillum in *Cassida piperata* (Cassidinae) and *Gastrophysa atrocyanea* (Chrysomelinae) as well as *G. grisescens* (Fig. S6). This result supports the idea that tarsal gustatory sensilla are detectable through SEM observation. Thus, SEM observations suggested that tarsal gustatory sensilla are common throughout Chrysomelidae. In contrast, no gustatory sensilla were found on the tarsi of *A. dichotoma*, *T. mucida*, *A. pictus*, and *H. vigintioctomaculata*. Although *H. vigintioctomaculata* is phytophagous like Chrysomelidae, we found no gustatory sensilla on their tarsi. It has been reported that the Mexican bean beetle, *Epilachna varivestis* Mulsant (Coleoptera; Coccinellidae) do not have gustatory sensilla on their tarsi^41^. Our results regarding the lack of gustatory sensilla in the three species from different beetle families, as well as in another coccinellid species, *H. vigintioctomaculata*, suggest that this character may not be common throughout Coleoptera. However, further studies should be carried out on other coleopteran families and on a wider range of species to fully understand the extent of this character in Coleoptera.

In Diptera and Lepidoptera, a single tarsal gustatory sensillum responds to several tastes, such as sweet and bitter^6,42^. In Coleoptera, salt reception through tarsi has been shown electrophysiologically^24^; however, sugar reception by tarsi has not been shown. Our result is the first evidence, to our knowledge, that some coleopteran insects can receive sugar through their tarsal gustatory sensilla. The tarsal gustatory sensilla of *G. grisescens* responded to salt, sucrose, brucine, and the leaf surface wax of *R. obtusifolius*. Thus, it was revealed that *G. grisescens* can perceive four basic tastes, which might be important in host selection using their tarsi. In our TEM observations, four dendrites were observed inside the sensillum lymph of tarsal gustatory sensilla of *G. grisescens* and *C. nebulosa*. It was suggested that the four gustatory neurons respond to different tastes, salt, sweet, bitter, and their host plant wax taste, respectively. In addition, the electrophysiological response of tarsal gustatory sensilla of *G. grisescens* was different depending on the location of the sensillum. For example, a sensillum located on the dorsal side of a claw (sensillum C1) showed no response to sucrose although it has four dendrites. In addition, only sensillum C2 responded to the leaf surface wax of *R. obtusifolius*. Thus, some sensilla did not show a response to some of the four taste substances tested. These sensilla might receive other taste substances not tested in this study as four dendrites were observed in all gustatory sensilla. From these results, it was assumed that the tarsal gustatory sensilla receive different taste substances according to location.

In the behavioural tests, *G. grisescens* and *C. piperata* chose sucrose tasted using only their tarsi (Figs. 6, S7). Conversely, *H. vigintioctomaculata* showed no response to sucrose tasted using only their tarsi, even though sweet taste is one of the basic tastes. From this result, we consider that *H. vigintioctomaculata* cannot taste through their tarsi. The ablation of the mouthparts seriously affected the taste recognition behaviour of *H. vigintioctomaculata*, indicating that the mouthparts play the most important role as gustatory organs in taste recognition in *H. vigintioctomaculata*. These differences might be related to the orientation of the mouthparts. The ventral side of the head of *H. vigintioctomaculata* is always oriented downward unlike those of Chrysomelidae, so that the mouthparts of *H. vigintioctomaculata* contact the plant surface as soon as the beetle reaches food plants. Thus, the gustatory organ that first touches the food surface might be different in both insects. It was assumed, therefore, that the presence of tarsal gustation is closely related to the location or direction of other gustatory organs, such as those on the mouthparts or antennae.

In the behavioural tests, *G. grisescens* individuals showed a positive response to their host plant leaf surface wax using only their tarsi. Conversely, they showed no response or a negative response to the leaf surface wax of nonhost plants, *S. melongena* and *S. oleracea*, which do not belong to the same family as *R. obtusifolius*. Furthermore, they showed no response to the leaf surface wax of the nonhost plant, *P. perfoliata*, which belongs to the same family as *R. obtusifolius*. These results suggest that *G. grisescens* can precisely discriminate their host plant from nonhost plants by using their tarsal gustatory organs. Taken together, it was revealed that *G. grisescens* can discriminate their host plant from nonhost plants through touching the leaf surface with their tarsi.

*cis*-3-Hexenyl acetate, a major volatile component of *R. obtusifolius*^27^, or other volatile substances, such as monoterpenes and sesquiterpenes, were not detected in the hexane extracts of *R. obtusifolius* analysed by coupled gas chromatography/mass spectrometry [GC/MS: Shimadzu GCMS-QP2010 Ultra, equipped with a DB-5MS column (30 m × 0.25 mm i.d., 0.25 µm film thickness, J&W, Santa Clara, CA, USA); carrier gas: helium; split ratio: 50:1; temperature program of the column oven: initial temperature 40 °C, 8 °C/min to 310 °C; injector temperature: 250 °C; detector temperature: 200 °C; interface temperatures: 300 °C. Mass spectral databases used for the analysis: Shimadzu GCMS Solution with the Wiley Registry (9th ed.), National Institute of Standards and Technology (NIST05), Flavor and Fragrance Natural and Synthetic Compounds (FFNSC ver. 1.3)].

In addition, although *G. grisescens* exhibited a positive response to leaf surface exudates of *R. obtusifolius* (extracted by dipping a leaf in ethanol for 30 s), they showed no response to the leaf surface exudate of *R. obtusifolius* after ablation of mouthparts, antennae or tarsi (Fig. S8). These results indicate that leaf surface wax components (ex. long-chain hydrocarbons and triterpenoid) elicited behavioural responses in *G. grisescens*. *G. grisescens* responded to alkanes and carboxylic acids of specific carbon numbers^28^; therefore, wax profiles might be important in host discrimination using tarsi. In our electrophysiological experiment using the leaf surface wax of *R. obtusifolius*, tarsal gustatory sensillum exhibited two different spikes. This result indicates that *G. grisescens* can detect at least two wax compounds.

In conclusion, we show that tarsal gustatory sensilla are common in Chrysomelidae. However, this character was not found in the species from the other coleopteran families that were examined. To clarify the generality of tarsal gustatory organs in Coleoptera, further observations are required. It was also indicated that tarsal taste recognition is important in the host selection process of *G. grisescens*. Moreover, behavioural response to sugar through tarsal taste recognition was shown in *C. piperata* as well as in *G. grisescens*. These morphological and behavioural investigations indicate that tarsal gustatory sensilla observed in chrysomelid species are assumed that they may play an important role in making decisions through taste, including host selection, in Chrysomelidae. Our results provide important information for better understanding the mechanisms of insect host selection. Agricultural pests are of particular interest because the host plants are crops used for human and livestock food. The results of our study regarding the family Chrysomelidae, a family containing many agricultural pests, are therefore important for the development of new insect behaviour regulators that act through their tarsi. In addition, we anticipate that our findings will provide new insights into taste recognition strategies corresponding to feeding habits in insects.

## Methods

### Insects

*Galerucella grisescens* individuals were reared on leaves of *R. obtusifolius* at a constant temperature of 24 ± 1 °C under a photoregime of 16 L:8D. The leaves of *R. obtusifolius* were collected from an experimental field at Tohoku University. Adult beetles (3- to 8-day-old) were used in the behavioural and electrophysiological experiments.

Adults of *Henosepilachna vigintioctomaculata* were collected from potato fields in Natori (Miyagi, Japan) and reared at a constant temperature of 24 ± 1 °C under a photoregime of 16 L:8D. The beetles were reared on tomato leaves collected from tomatoes cultivated in the experimental field or greenhouse of Tohoku University. Adult beetles (4- to 11-day-old) were used in the experiments.

*Cassida piperata* individuals were reared on leaves of *Chenopodium album* var. centrorubrum at a constant temperature of 24 ± 1 °C under a photoregime of 16 L:8D. The leaves of *Chenopodium album* var. centrorubrum were collected from an experimental field at Tohoku University. In the behavioural experiments, 4- to 7-day-old adult beetles were used, and 4- to 14-day-old adult beetles were used in the electrophysiological experiments.

*Gastrophysa atrocyanea* individuals were collected from an experimental field at Tohoku University. The beetles were reared on leaves of *R. obtusifolius* at a constant temperature of 24 ± 1 °C under a photoregime of 16 L:8D. The leaves of *R. obtusifolius* were collected from an experimental field at Tohoku University, and 3- to 10-day-old adult beetles were used in the electrophysiological experiments.

Beetles observed under SEM are listed in Table S1.

### Extraction of leaf surface wax and leaf surface exudate

The leaf surface waxes were extracted from leaves of *R. obtusifolius*, *S. melongena*, *S. oleracea*, and *P. perfoliata*. Intact leaves were collected from either the experimental field or a greenhouse at Tohoku University where they had been cultivated at 20 °C. The total surface area of the collected leaves of each plant was calculated before extraction. The collected leaves were individually dipped into 800 mL of hexane for 30 s. During the extraction, the cut end of a leaf was not allowed to touch the solvent. Thereafter, extracts were filtered through filter paper (ADVANTEC^®^, No.1, Toyo Roshi Kaisha, Ltd., Tokyo, Japan) and evaporated at 38 °C using a rotary vacuum evaporator to obtain leaf surface waxes. The results of the leaf surface wax extraction are shown in Table S2.

The leaf surface exudates were extracted from leaves of *R. obtusifolius*. Intact leaves were collected from either the experimental field or a greenhouse at Tohoku University where they had been cultivated at 20 °C. The total surface area of the collected leaves of each plant was calculated before extraction. The collected leaves were individually dipped in 800 mL of methanol for 30 s. During the extraction, the cut end of a leaf was not allowed to touch the solvent. Thereafter, extracts were filtered through filter paper (ADVANTEC^®^, No.1, Toyo Roshi Kaisha, Ltd., Tokyo, Japan) and evaporated at 38 °C using a rotary vacuum evaporator to obtain leaf surface exudate.

### Scanning electron microscopy

The tarsi of beetles were removed from the legs and fixed onto a SEM platform with a double-sided carbon tape. The tarsi were then desiccated and coated with platinum-palladium. Samples were observed using a SEM (SU8000type II, Hitachi Ltd., Tokyo, Japan). The tarsi of 49 species of Chrysomelidae (Table S1) as well as of *A. dichotoma*, *T. mucida*, *A. pictus*, and *H. vigintioctomaculata* were observed.

### Transmission electron microscopy

Tarsi were removed from the legs of *G. grisescens* and fixed in 2.5% glutaraldehyde in 0.2 M phosphate buffer solution (PBS) (pH = 7.0) overnight in a refrigerator. The tarsi were then rinsed three times with PBS and fixed in 1% osmium tetroxide dissolved in PBS for 2 h. Following fixation, the tarsi were dehydrated with a series of different concentrations of ethanol (50–100%). Subsequently, the tarsi were treated with propylene oxide to replace the solvent and then embedded in an epoxy resin by changing the ratio of propylene oxide and epoxy resin. Ultra-thin sections of 80 nm were cut on an ultra-microtome using a diamond knife and collected on formvar-coated copper grids. The grids were observed with a TEM (H-7650 Zero A, Hitachi Ltd.).

### Electrophysiological recording of the response of tarsal gustatory sensilla to tastants

A tip-recording method was used to record the responses of a single sensillum to five stimulants, namely KCl, NaCl, sucrose, brucine, and the leaf surface wax of *R. obtusifolius*. Recordings were obtained from 3- to 8-day-old-adult male and female *G. grisescens* individuals. Beetles were mounted on a kneaded eraser with their ventral side up after their elytra, mid-legs, and hind-legs were removed. A glass capillary filled with 100 mM KCl connected to the ground was inserted into the thorax as an indifferent electrode. Three tarsal gustatory sensilla on the 5^th^ tarsomere (Fig. S3) were tested. Each gustatory sensillum was stimulated by covering its tip with a glass micro pipette filled with a solution of the stimulant for 3 s. Sensillum response was recorded from the onset of stimulation. To minimize the adaptation of the tested sensilla, an interval of at least 3 min was allowed between consecutive recordings^6^. Action potentials generated by gustatory sensilla were amplified by Taste Probe DTP-1 (Syntech, Hilversum, Netherlands) and filtered using IDAC-4 (Syntech, Hilversum, Netherlands). Data were analysed using the AutoSpike v3.7 software (Syntech, Hilversum, The Netherlands). Neural response was measured by doubling the number of spikes generated in 200–700 ms after the onset of recording. Owing to its hydrophobicity, 1% Tween 20 in distilled water was used to dissolve the leaf surface wax of *R. obtusifolius*. All the tested solutions contained 5 mM of KCl as an electrolyte except those used for recording the responses to KCl and NaCl.

### Two-choice assays

Half-disk assays were used to investigate whether leaf beetles could behaviourally respond to taste substances using only their tarsi. The behavioural responses of *G. grisescens* to sucrose, brucine, and leaf surface waxes of host and nonhost plants were investigated. For comparison, the behavioural response of *H. vigintioctomaculata* and *C. piperata* to sucrose was also investigated. A sheet of filter paper (ADVANTEC^®^, No.1, 90 mm diameter, Toyo Roshi Kaisha, Ltd., Tokyo, Japan) was cut in the middle and one of the two half-filter-paper disks was treated with a test substance solution (treated half-disk). The other was used as a control (control half-disk). These half-disks were joined edge-to-edge with scotch tape on the bottom side after the solvent was completely evaporated. To prevent the half-disks from coming into contact with each other, a 1-mm gap was provided between them. A circular disk recomposed from the half-disks was placed on the bottom of the lid of a glass Petri dish (90 mm diam.) (Fig. S9). To increase the humidity, 400 µL of pure water was added to each half-disk.

To investigate which gustatory organ is preferentially used for taste recognition, we ablated the mouthparts (labial palpi and maxillary palpi), antennae, and tarsi of beetles in different combinations. Ablations were conducted using precision tweezers under a stereo microscope. As male and female individuals showed similar behavioural and electrophysiological response to sucrose, brucine, and leaf surface wax of their host plant, *R. obtusifolius* (Figs. 4, S10,S11), unsexed beetles were used in experiments. Beetles were starved for 24 h before the experiments and released on each half-disk immediately before the start of the experiment. The inner wall of the Petri dish was coated with a polishing agent to prevent the beetles from climbing. After releasing the insects, a lid, with its base inverted, was placed over the Petri dish. Experiments were conducted at 25 °C in the dark condition. After 1 h, the number of beetles on each half-disk was counted. In *G. grisescens*, 20 individuals were used in each replication. In *H. vigintioctomaculata* and *C. piperata*, 10 individuals were used in each replication. We investigated the responses of *H. vigintioctomaculata*, in which we did not find tarsal gustatory sensilla under SEM or TEM, to confirm whether the results of our morphological observation corresponded to the behavioural response of the tarsi. We used sucrose because this sugar is a strong feeding stimulant for *H. vigintioctomaculata*^38,39^ and is important for them to select a feeding site.

In the test for response to sucrose, treated half-disks were impregnated with 400 µL of sucrose solution (500 mM in distilled water) and the control half-disks were impregnated with 400 µL of distilled water. In the test for response to brucine, 318 µL of a solution of brucine (100 mg/mL) in methanol was applied to the treated half-disks as the tastant and 318 µL of methanol was applied to the control half-disks. In the test for response to leaf surface waxes, 318 µL of a solution of leaf surface wax in hexane was applied to the treated half-disks. Control half-disks were treated with 318 µL of hexane. The dose (µg/cm^2^) of the leaf surface wax applied to the treated half-disks was equivalent to the mean quantity of the wax contained in 1 cm^2^ of leaf of each plant tested. In the test for response to leaf surface exudates, 318 µL of a solution of leaf surface exudates in hexane was applied to the treated half-disks. Control half-disks were treated with 318 µL of methanol. The dose (µg/cm^2^) of the leaf surface exudates applied to the treated half-disks was equivalent to the mean quantity of the exudates contained in 1 cm^2^ of leaf of each plant tested.

### Statistical analysis

The results of two-choice assays were analysed using the Wilcoxon matched-pairs signed-ranks test. Statistical analyses were performed using R version 3.5.1^43^.

## Supplementary information

Supplementary information

## Data Availability

The datasets are available from the corresponding author on reasonable request.
