# Supplementary material for: Taste recognition through tarsal gustatory sensilla potentially important for host selection in leaf beetles (Coleoptera: Chrysomelidae)
Source: Sci Rep. 2020 Mar 18;10:4931. doi: 10.1038/s41598-020-61935-x (PMC7080798; doi:10.1038/s41598-020-61935-x)
Supplement: Supplementary file 1 — Supplementary information [file 41598_2020_61935_MOESM1_ESM.docx]

**Taste recognition through tarsal gustatory sensilla potentially important for host selection in leaf beetles (Coleoptera: Chrysomelidae)**

Shun Yosano^1^, Yasuhiko Kutsuwada^1^, Minami Akatsu^1^, Shuhei Masuta^1^, Rei Kakazu^1^, Naoshi Masuoka^1^, Kazuhiro Matsuda^1^, Masatoshi Hori^1*^

^1^Graduate School of Agricultural Science, Tohoku University, Sendai, Miyagi 980-8572, Japan

^*^Correspondence: hori@tohoku.ac.jp


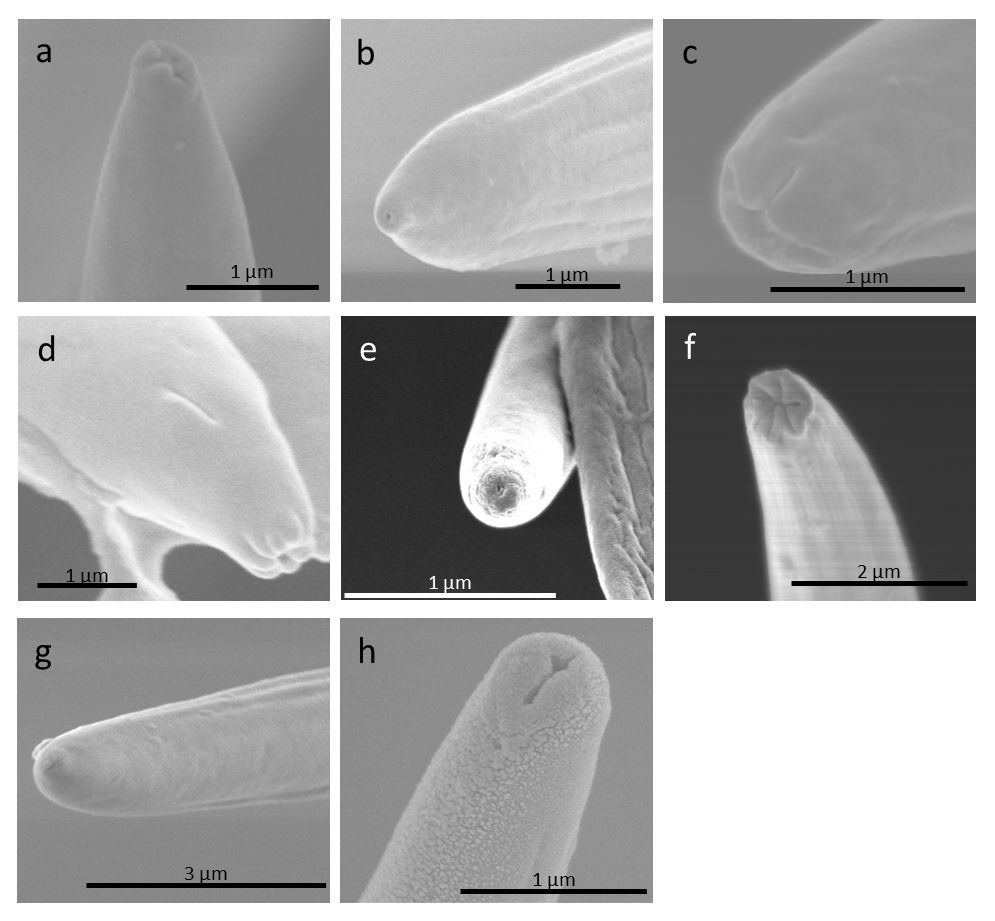


**Supplementary Figure S1.** Tarsal gustatory sensilla of Chrysomelidae 1

Tarsal gustatory sensilla of *Colobaspis japonica* (Megalopodinae). (b) Tarsal gustatory sensilla of *Cassida nebulosa* (Cassidinae). (c) Tarsal gustatory sensilla of *Lilioceris subpolita* (Criocerinae). (d) Tarsal gustatory sensilla of *Sagra femorata* (Sagrinae). (e) Tarsal gustatory sensilla of *Chlamisus laticollis* (Chlamisinae). (f) Tarsal gustatory sensilla of *Demotina fasciculata* (Eumolpinae). (g) Tarsal gustatory sensilla of *Cryptocephalus approximatus* (Cryptocephalinae). (h) Tarsal gustatory sensilla of *Oomorphoides cupreatus* (Lamprosomatinae).


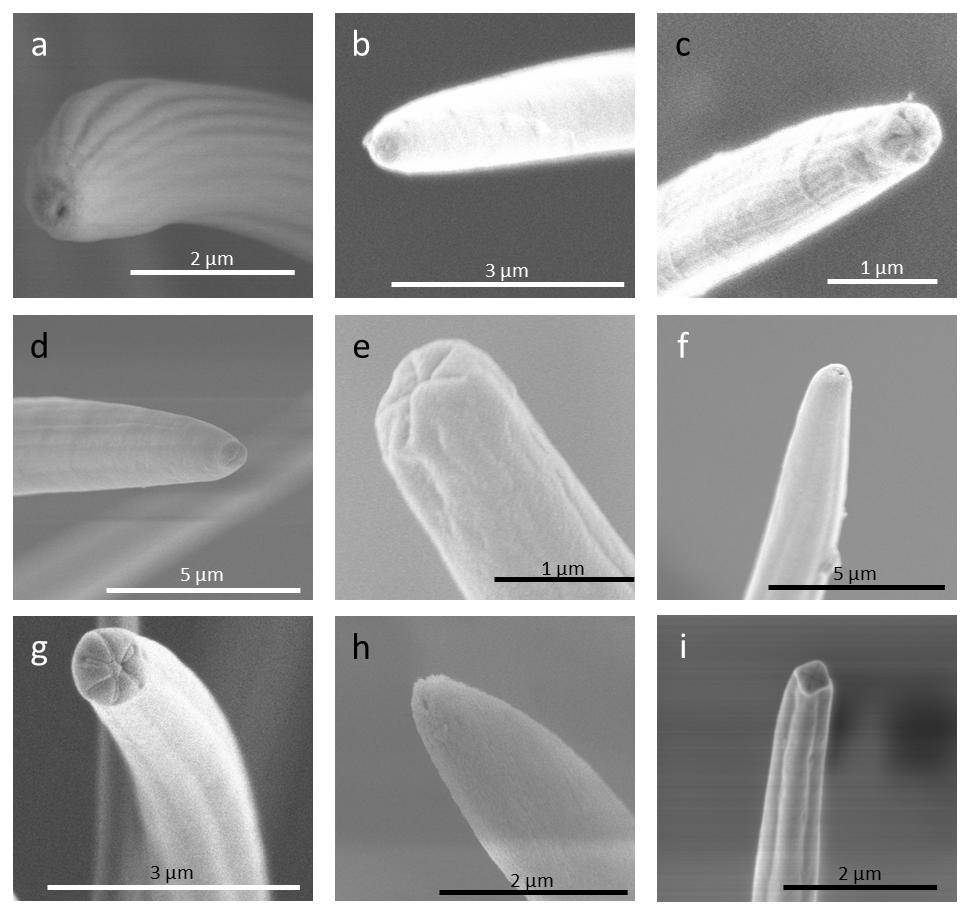
**Supplementary Figure S2.** Tarsal gustatory sensilla of Chrysomelidae 2.

(a) Tarsal gustatory sensilla of *Dactylispa subquadrata* (Hispinae). (b) Tarsal gustatory sensilla of *Smaragdina aurita* (Clytrinae). (c) Tarsal gustatory sensilla of *Orsodacne arakii* (Orsodacnidae). (d) Tarsal gustatory sensilla of *Plateumaris constricticollis babai* (Donaciinae). (e) Tarsal gustatory sensilla of *Psedodera xanthospila* (Alticinae). (f) Tarsal gustatory sensilla of *Gastrolina depressa* (Chrysomelinae). (g) Tarsal gustatory sensilla of *Galerucella grisescens* (Galerucinae). (h) Tarsal gustatory sensilla of *Syneta adamsi* (Synetinae). (i) Tarsal gustatory sensilla of *Callosobruchus chinensis* (Brucinae).


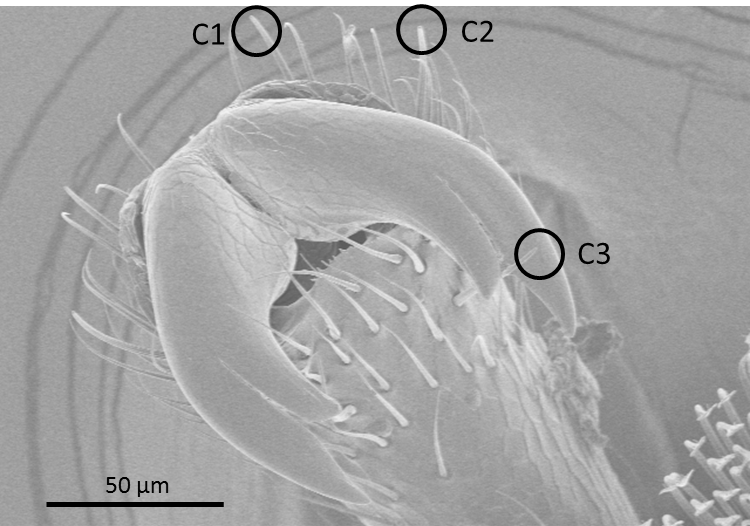


**Supplementary Figure S3.** Location of tarsal gustatory sensilla of *Galerucella grisescens*.

Location of three tarsal gustatory sensilla (C1–C3) which were investigated electrophysiologically.


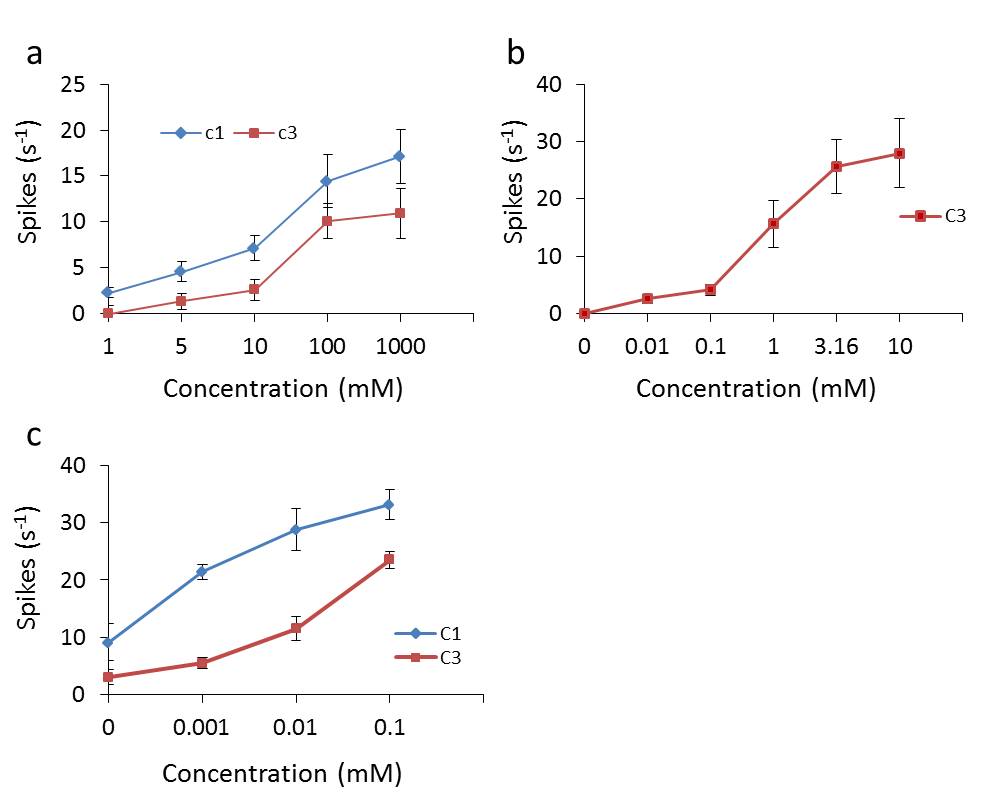


**Supplementary Figure S4.** Electrophysiological response of sensillum C1 and sensillum C3. (a – c) Electrophysiological responses of tarsal gustatory sensilla of *Galerucella grisescens*. Mean spike frequencies generated by applying solution of taste substances of each concentration are shown. (a) Electrophysiological response of sensilla C1 and C3 to KCl (C1: n = 7; C3: n =12). (b) Electrophysiological response of sensillum C3 to sucrose (n = 12). (c) Electrophysiological response of sensilla C1 and C3 to brucine (C1: n = 7; C3: n =12).


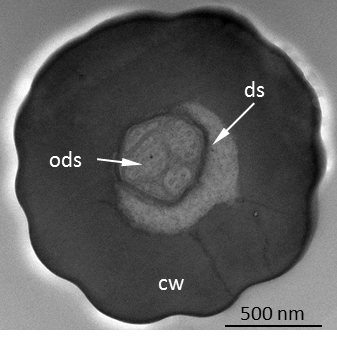


**Supplementary Figure S5.** Cross section of a tarsal sensillum chaeticum of *Cassida nebulosa* at the peripheral level. Four dendrites are enveloped by a dendritic sheath. ods: outer dendritic segment. ds: dendritic sheath. cw: cuticular wall.


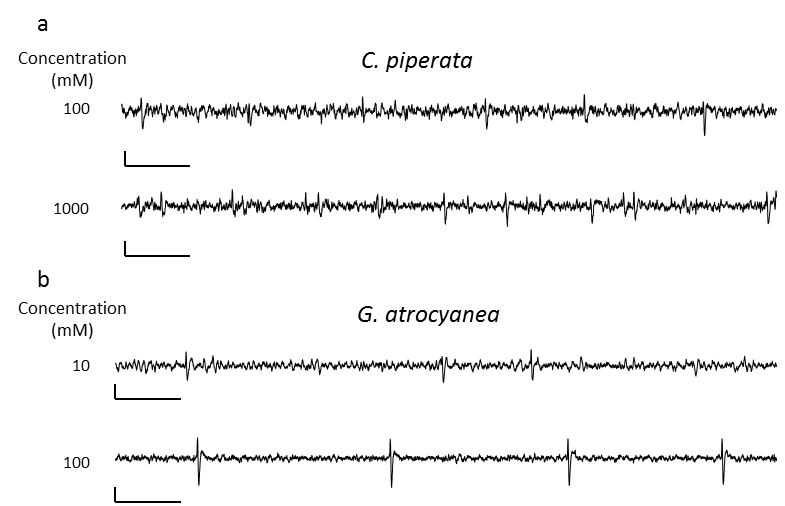


**Supplementary Figure S6.** Representative traces of electrophysiological responses obtained from the tarsal gustatory sensilla of *Cassida piperata and Gastrophysa atrocyanea.* Electrophysiological responses to KCl were obtained using the tip-recording method. (a) Electrophysiological response of *C. piperata* tarsal gustatory sensillum to KCl (vertical bar, 400 µV; horizontal bar, 50 ms). (b) Electrophysiological response of *C. piperata* tarsal gustatory sensillum to KCl (vertical bar, 1 mV; horizontal bar, 50 ms).


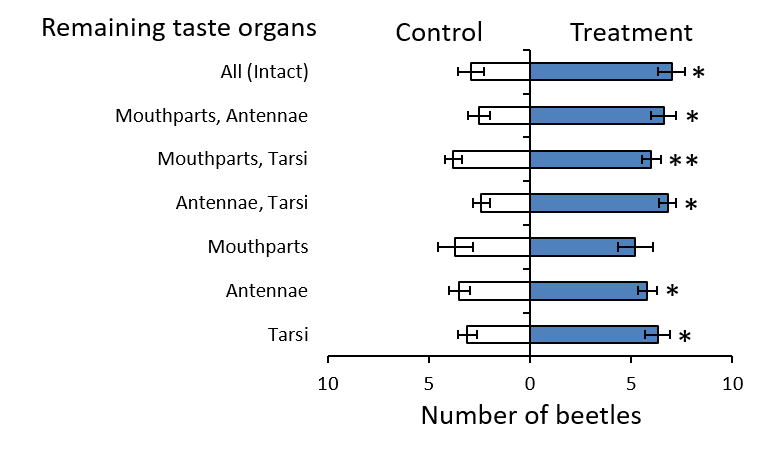


**Supplementary Figure S7.** Response of *Cassida piperata* to sucrose. Results of two choice tests for *C. piperata* (10 beetles in each test; n = 10). Vertical line shows the gustatory organ that remained after ablation. Horizontal axis shows the mean number of beetles (± SE) that chose the treated or the control half-disk. A significant difference between the treatment and control is represented by an asterisk (Wilcoxon matched-pairs signed-ranks test: * *p* < 0.05).


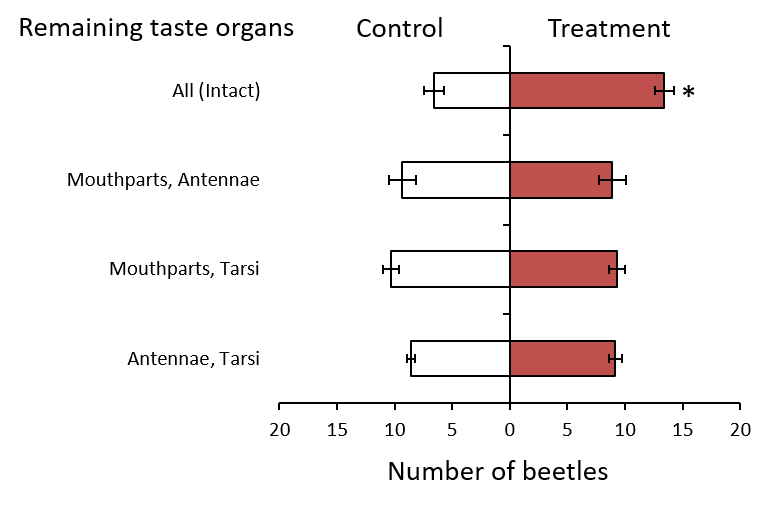


**Supplementary Figure S8.** Response of *Galerucella grisescens* to leaf surface exudate of *Rumex obtusifolius*. Results of two choice tests for *G. grisescens* (20 beetles in each test; n = 12). Vertical line shows the gustatory organ that remained after ablation. Horizontal axis shows the mean number of beetles (± SE) that chose the treated or the control half-disk. A significant difference between the treatment and control is represented by an asterisk (Wilcoxon matched-pairs signed-ranks test: * *p* < 0.05, ** *p* < 0.01).


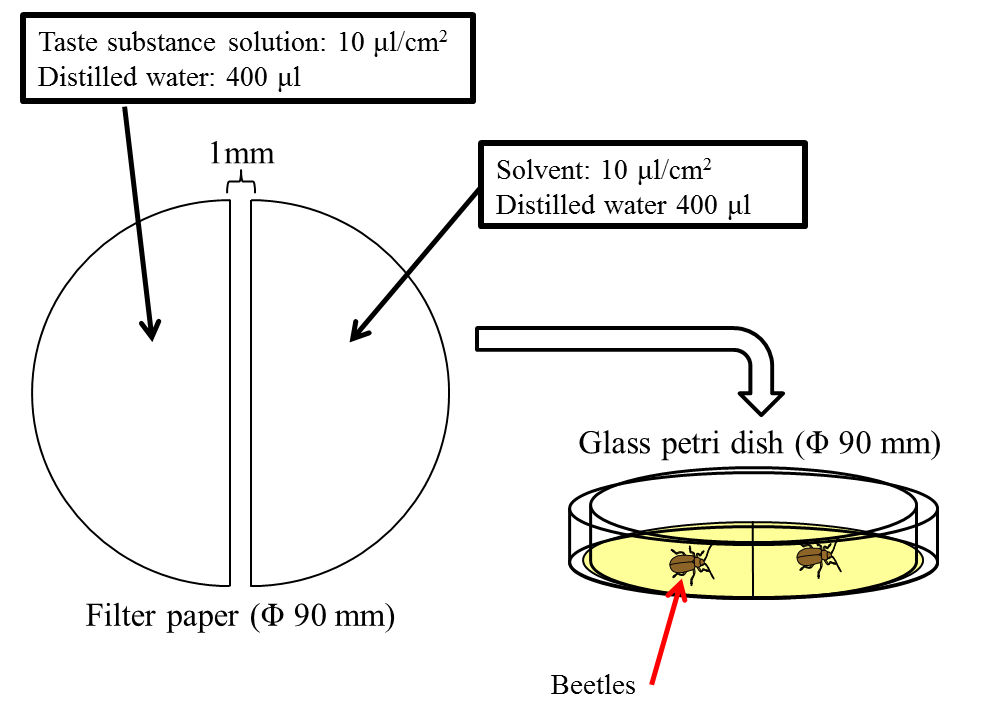


Supplementary Figure S9. Two choice assays using the half-disk method.


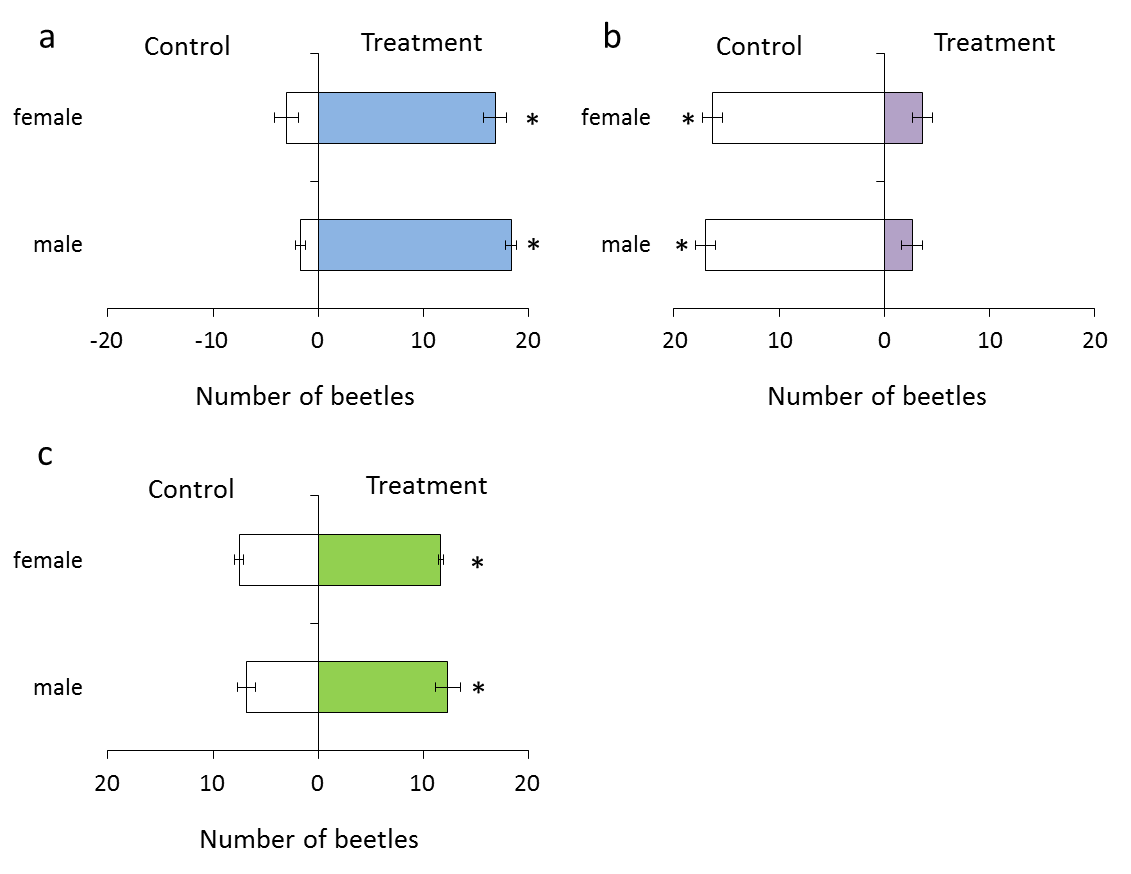


**Supplementary Figure S10.** Response of *Galerucella grisescens* to sucrose, brucine, and leaf surface wax of *Rumex obtusifolius*. Results of two choice tests for male and female *G. grisescens*. (a–c) Vertical line shows the gustatory organ that remained after ablation. Horizontal axis shows the mean number of beetles (± SE) that chose the treated or the control half-disk. (a) Response of *G. grisescens* to sucrose (20 beetles in each test; n = 6). (b) Response of *G. grisescens* to brucine (20 beetles in each test; n = 6). (c) Response of *G. grisescens* to leaf surface wax of *R. obtusifolius* (20 beetles in each test; n = 6). A significant difference between the treatment and control is represented by an asterisk (Wilcoxon matched-pairs signed-ranks test: * *p* < 0.05).


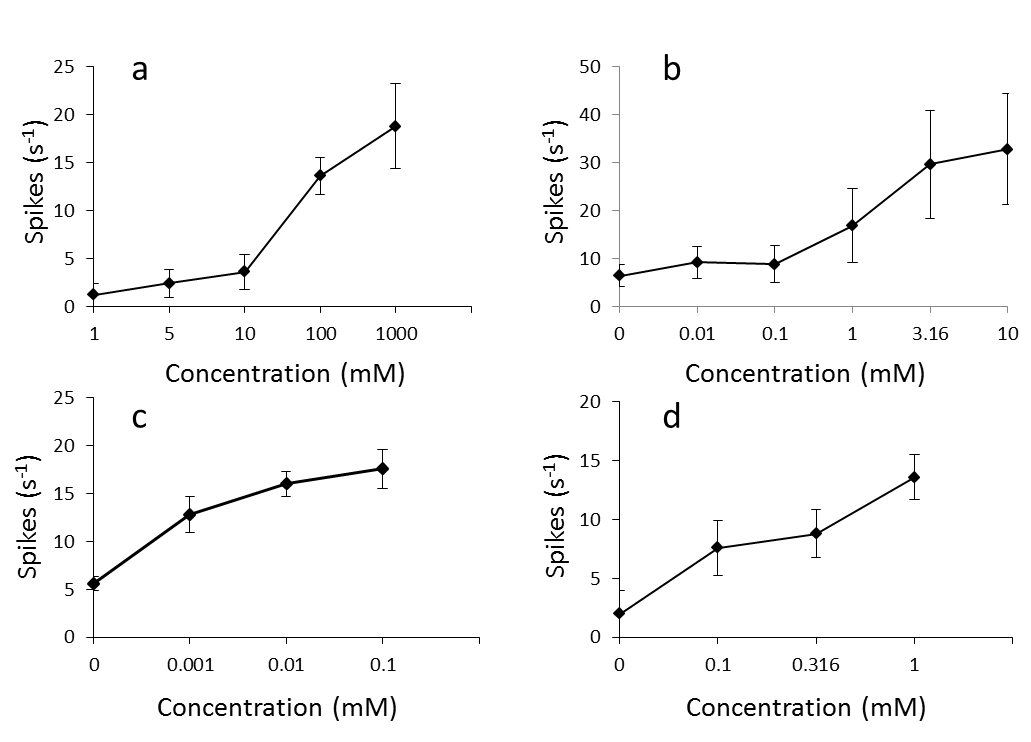


**Supplementary Figure S11.** Taste reception by tarsal gustatory sensilla of male *Galerucella grisescens.* (a–d) Electrophysiological responses of tarsal gustatory sensilla of male *G. grisescens.* All recordings were obtained from sensillum C2 using the tip-recording method. Mean spike frequencies (± standard error) generated by applying a solution of each taste substance at each concentration are shown. (a) Responses to KCl. (b) Response to sucrose. (c) Response to brucine. (d) Response to the leaf surface wax of *R. obtusifolius*. Data were obtained from 5 beetles for each tastant.

Supplementary Table S1. List of coleopteran species observed with SEM.

| Family | Subfamily | Specie | Number of observed specimens | Collection area |
| --- | --- | --- | --- | --- |
| Chrysomelidae | Megalopodinae | *Colobaspis japonicas* | 1 | Miyagi, Japan |
|  |  | *Zeugophora annulate* | 1 | Miyagi, Japan |
|  | Brucinae | *Callosobruchus chinensis* | 4 | Beetles were obtained from a continuous culture reared in our laboratory. |
|  | Sagrinae | *Sagra femorata* | 3 | Mie, Japna |
|  | Donaciinae | *Donacia hiurai* | 1 | Miyagi, Japan |
|  |  | *Plateumaris constricticollis babai* | 4 | Miyagi, Japan |
|  | Criocerinae | *Lema honorata* | 1 | Miyagi, Japan |
|  |  | *Lilioceris subpolita* | 2 | Fukushima, Japan |
|  | Orsodacnidae | *Orsodacne arakii* | 3 | Yamanashi, Japan |
|  | Synetinae | *Syneta adamsi* | 8 | Miyagi, Japan |
|  | Eumolpinae | *Acrothinium gaschkevitchii* | 2 | Miyagi, Japan |
|  |  | *Basilepta balyi* | 1 | Miyagi, Japan |
|  |  | *Demotina fasciculata* | 1 | Miyagi, Japan |
|  |  | *Trichochrysea japana* | 2 | Miyagi, Japan |
|  | Galerucinae | *Agelasa nigriceps* | 2 | Miyagi, Japan |
|  |  | *Atrachya menetriesi* | 3 | Miyagi, Japan |
|  |  | *Aulacophora nigripennis* | 4 | Miyagi, Japan |
|  |  | *Fleutiauxia armata* | 5 | Miyagi, Japan |
|  |  | *Galeruca extensa* | 1 | Miyagi, Japan |
|  |  | *Gallerucida bifasciata* | 2 | Miyagi, Japan |
|  |  | *Galerucella grisescens* | 21 | Beetles were obtained from a continuous culture reared in our laboratory. |
|  |  | *Pyrrhalta humeralis* | 2 | Miyagi, Japan |
|  | Alticinae | *Altica latericosta* | 2 | Miyagi, Japan |
|  |  | *Argopistes biplagiata* | 3 | Miyagi, Japan |
|  |  | *Philopona vibex* | 1 | Miyagi, Japan |
|  |  | *Pseudodera xanthospila* | 2 | Miyagi, Japan |
|  | Chrysomelinae | *Chrysolina exanthematica* | 7 | Miyagi, Japan |
|  |  | *Gastrolina depressa* | 4 | Miyagi, Japan |
|  |  | *Gastrophysa altrocyanea* | 5 | Miyagi, Japan |
|  |  | *Gonioctena rubripennis* | 3 | Miyagi, Japan |
|  |  | *Linaeidea aenea* | 1 | Miyagi, Japan |
|  | Cassidinae | *Aspidomorpha indica* | 3 | Miyagi, Japan |
|  |  | *Cassida erudite* | 2 | Miyagi, Japan |
|  |  | *Cassida nebulosi* | 5 | Beetles were obtained from a continuous culture reared in our laboratory. |
|  |  | *Cassida piperata* | 27 | Beetles were obtained from a continuous culture reared in our laboratory. |
|  |  | *Cassida rubiginosa* | 4 | Miyagi, Japan |
|  |  | *Cassida versicolor* | 9 | Miyagi, Japan |
|  |  | *Thlaspida cribrosa* | 5 | Miyagi, Japan |
|  |  | *Thlaspida lewisii* | 2 | Miyagi, Japan |
|  | Hispinae | *Brontispa longissimi* | 6 | Beetles were provided from National Agriculture and Food Research Organization (Japan). |
|  |  | *Dactylispa angulosa* | 4 | Miyagi, Japan |
|  |  | *Dactylispa subquadrata* | 4 | Miyagi, Japan |
|  | Iampososomatinae | *Oomorphoides cupreatus* | 2 | Fukushima, Japan |
|  | Chlamisinae | *Chlamisus Laticollis 1* | 19 | Miyagi, Japan |
|  | Cryptocephalinae | *Cryptocephalus approximatus* | 1 | Miyagi, Japan |
|  |  | *Cryptocephalus fortunatus* | 1 | Miyagi, Japan |
|  |  | *Cryptocephalus signaticeps* | 1 | Miyagi, Japan |
|  | Clytrinae | *Smaragdina aurita* | 4 | Gunma, Japan |
|  |  | *Smaragdina semiaurantiaca* | 1 | Nagano, Japan |
| Coccinellidae |  | *Henosepilachna vigintioctomaculata* | 8 | Miyagi, Japan |
| Scarabaeidae |  | *Allomyrina dichotoma* | 4 | Miyagi, Japan |
| Cupedidae |  | *Tenomerga mucida* | 1 | Miyagi, Japan |
| Endomychidae |  | *Ancylopus pictus* | 3 | Miyagi, Japan |

Supplementary Table S2. Results of leaf surface extraction.

| Plant | Leaf surfce area (cm^2^) | Amount of obtained wax (μg) | Wax per unit area (μg/cm^2^) | Date |
| --- | --- | --- | --- | --- |
| *Rumex obtusifolius* | 2614.111 | 71200 | 27.24 | 2013.7.19 |
| *Rumex obtusifolius* | 2923.245 | 84700 | 28.97 | 2013.7.22 |
| *Solanum melongena*(Shikonsendainaga) | 241.4888 | 7900 | 32.71 | 2015.2.8 |
| *Solanum melongena*(Shikonsendainaga) | 268.5913 | 7000 | 26.06 | 2015.2.10 |
| *Spinacia oleracea* (Ajimidori) | 2059.31 | 60000 | 29.14 | 2017.6.25 |
| *Spinacia oleracea* (Ajimidori) | 1854.881 | 16500 | 8.90 | 2018.6.22 |
| *Persicaria perfoliata* | 2690.86 | 85300 | 31.70 | 2015.8.18 |
